# Efficacy and safety of first-line immunotherapy-containing regimens compared with chemotherapy for advanced or metastatic urothelial carcinoma: a network meta-analysis of randomized controlled trials

**DOI:** 10.3389/fonc.2024.1453338

**Published:** 2024-12-11

**Authors:** Weiming Liang, Shibo Huang, Yanping Huang, Miaoyan Huang, Chunyan Li, Yiwen Liang, Li Pang

**Affiliations:** ^1^ The First Affiliated Hospital of Guangxi University of Science and Technology, Guangxi University of Science and Technology, Liuzhou, Guangxi, China; ^2^ Medicine Center, Guangxi University of Science and Technology, Liuzhou, Guangxi, China

**Keywords:** uc, immunotherapy, immune-checkpoint inhibitor, enfortumab vedotin, PD-1 inhibitor, chemotherapy, first-line, meta-analysis

## Abstract

**Introduction:**

To assess the efficacy and safety of first-line immunotherapy-containing regimens compared with chemotherapy for advanced or metastatic urothelial carcinoma (UC).

**Method:**

A comprehensive search was performed in four databases (Pubmed, Embase, Web of Science, and the Cochrane Library) to identify randomized controlled trials (RCTs) assessing the efficacy of first-line immunotherapy-containing regimens for advanced or metastatic UC. The search encompassed the time span from the inception of the databases to April 23, 2024. A network meta-analysis (NMA) was conducted to assess the rates of progression-free survival (PFS), overall survival (OS), complete response (CR), objective response rate (ORR), and grade ≥ 3 adverse events (AEs).

**Results:**

We conducted a comprehensive analysis of five randomized controlled trials (RCTs) that included a total of 4749 patients. Nine different treatment regimens included in the study were ranked statistically and intuitively using NMA. The top five effective regimens, ranked by OS, were EV + Pembro (1.000), Nivol + Chemo (0.724), Atezo + Chemo (0.610), Durva + Treme (0.558), and Pembro + Chemo (0.530). The top five effective regimens, ranked by PFS, were EV + Pembro (0.999), Nivol + Chemo (0.640), Pembro + Chemo (0.484), Atezo + Chemo (0.373) and Chemo (0.003). The top five effective regimens, ranked by CR, were EV + Pembro (0.969), Nivol + Chemo (0.803), Atezo + Chemo (0.772), Pembro + Chemo (0.472), Durva + Treme (0.449). The top five effective regimens, ranked by ORR, were EV + Pembro (0.995), Nivol + Chemo (0.852), Pembro + Chemo (0.761), Atezo + Chemo (0.623), and Chemo (0.519).

**Conclusion:**

Our results indicated that EV + Pembro as first-line therapy resulted in considerably improved efficacy and safety compared to chemotherapy for advanced or metastatic UC. ICI plus chemotherapy as first-line treatment resulted in a longer PFS, a greater ORR, but no longer OS compared to chemotherapy alone, as well as higher toxicity. ICI alone as first-line therapy provided similar OS and lower toxicity compared to chemotherapy, but lower ORR.

**Systematic review registration:**

https://www.crd.york.ac.uk/prospero, identifier CRD42024538546.

## 1 Introduction

urothelial carcinoma (UC) is a prevalent form of genitourinary cancer and is considered one of the most widespread and lethal malignancies globally (1, 2). Tumors can manifest in any part of the genitourinary system, encompassing the urethra, bladder (which is the predominant location, accounting for over 90% of cases), ureters, and renal pelvis (3). The majority of patients present with early and potentially curable disease at diagnosis, with 10-15% experiencing progression of the disease to an invasive form (4). The standard therapy for muscle-invasive UC (MIUC) is either radical cystectomy (RC) or nephroureterectomy, along with neoadjuvant chemotherapy (NAC). The prognosis for individuals with advanced or metastatic UC is bleak, as more than 90% of these patients succumb to metastatic illness within a span of 5 years (5–7).

Before the introduction of immunotherapy, patients with refractory UC had limited follow-up choices, which included single-agent chemotherapeutic drugs like vincristine and docetaxel, or optimal supportive care. Unfortunately, the OS rate was low (8, 9). Response rates varied between 40% and 60%, resulting in a median OS of about 15 months for patients with metastatic UC. However, the long-term therapeutic benefit was not excellent, as only a small number of patients lived beyond 24 months (10). While cisplatin-based chemotherapy has enhanced the survival rates of patients with UC, the occurrence of relapse continues to be frequent (11). In the last five years, immune checkpoint inhibition has become a viable treatment for patients with metastatic UC. Four immune checkpoint inhibitors (ICIs) have been licensed for this condition, namely pembrolizumab and nivolumab, which are PD-1 inhibitors, and atezolizumab and avelumab, which are PD-L1 inhibitors (3). Nonetheless, there is still a need for a new agent that, when used in conjunction with platinum-based chemotherapy, can increase survival rates in the first-line treatment of metastatic UC. Although platinum chemo + nivolumab has become current frontline standard of care regimens for metastatic UC in the US based on the phase 3 trials, other clinical trials that have examined combinations of chemotherapy and other immune checkpoint inhibition in patients with locally advanced or metastatic UC have not demonstrated an improvement in OS (12–14). Besides, the EV-302 study findings demonstrated that the administration of enfortumab vedotin plus pembrolizumab yielded significantly superior results compared to chemotherapy in individuals diagnosed with untreated locally advanced or metastatic UC (15). Since the different outcomes of first-line immunotherapy-containing regimens reported in recent trials (16), it is necessary to perform a NMA to compare the efficacy and safety of these regimens.

In this network meta-analysis, we aimed to systematically evaluate the safety and efficacy of first-line immunotherapy compared with chemotherapy in patients with advanced or metastatic UC.

## 2 Materials and methods

### 2.1 Search strategy

The present meta-analysis was performed in accordance with the 2020 standards of the Preferred Reporting Project for Systematic Review and Meta-Analysis (PRISMA). This study has been registered at PROSPERO with a registration number of CRD42024538546. A search was conducted using a combination of MeSH terms and free-text words according to the PICOS principle. A comprehensive search was conducted on the electronic databases PubMed, Cochrane Library, Embase, and Web of Science. The search method employed was as follows: “urothelial carcinoma” AND “immunotherapy” AND “randomized controlled trial”. **Supplementary Material 1** presented the searching record in detail.

### 2.2 Inclusion and exclusion criteria

Inclusion criteria were as follows: (1) patients with untreated metastatic or advanced UC; (2) the intervention group was administered immunotherapy as first-line therapy, with or without other therapy; (3) the controlled group was administered chemotherapy as first-line therapy; (4) at least one of the following results were documented: CR, ORR, OS, PFS and grade ≥ 3 AEs (5);Types of studies: RCTs.

The exclusion criteria are as follows: (1) other types of articles, such as reviews, case reports, animal experimental studies, letters to editor, conference abstracts, comments, etc; (2) other cancers or diseases; (3) not relevant; (4) not first-line therapy; (5) single-arm studies; (6) failed to extract data; (7) duplicate patient cohort.

### 2.3 Selection of studies

The process of literature selection, which involved removing duplicate entries, was conducted using EndNote (Version 20; Clarivate Analytics).The initial search was carried out by two separate reviewers. The duplicate data was eliminated, and the relevance of the titles and abstracts was assessed to classify each study as either included or excluded. We resolved the issue by reaching a consensus. If the parties were unable to reach an agreement, a third reviewer took on the role of a mediator.

### 2.4 Data extraction

The data was extracted independently by two reviewers. The obtained data included crucial study details, such as the main author, year of publication, nation, research technique, sample size, and principal results. The study initially considered the demographic details of the participants, including the number of patients, their age, and the type of tumor they had. The data analyzed in the study included various measures such as Kaplan-Meier (KM) curves for OS, KM curves for PFS, CR, ORR, Grade ≥ 3 AEs. The issue was settled by seeking counsel from a third investigator.

### 2.5 Risk of bias assessment

Two independent reviewers evaluated the risk of bias in the included studies using the Cochrane Risk of Bias tool. This tool consists of seven domains: (1) random sequence generation, (2) allocation concealment, (3) blinding of participants and personnel, (4) blinding of outcome assessment, (5) incomplete outcome data, (6) selective reporting, and (7) other biases. In the event of any inconsistencies, the contentious outcomes were resolved by collective deliberation. In the event of any inconsistencies, the conflicting conclusions were resolved through collective discussion.

### 2.6 Statistical analysis

The process of removing duplicate studies from the selection was performed using EndNote (Version 20; Clarivate Analytics). The data analysis for this study was conducted using Review Manager 5.3 from the Cochrane Collaboration in Oxford, UK, as well as the statistical software R (version 4.3.1) obtained from https://www.r-project.org/. Specifically, the R package “netmeta” was utilized for the data analysis. For all meta-analyses, the Cochrane Q p value and I^2^ statistic were applied to check heterogeneity. Pooled data were analyzed using a fixed-effect model if heterogeneity was low or moderate (I^2^ <50%), or a random-effect model if heterogeneity was high (I^2^ ≥50%). Our study employed a frequency-based approach to assess the effectiveness of direct and indirect therapies, with chemotherapy serving as the common reference group (17). The assessment for OS, PFS, CR, ORR and Grade ≥ 3 AEs utilized contrast-based methods, where estimated differences in the logarithm of the hazard ratio (HR) and the standard error were determined based on the reported HR and confidence interval (CI) (18). The treatment effects were expressed as HR along with their corresponding 95% credible intervals (CrI). The network meta-analysis of dichotomous variables yielded results in the form of odds ratios (OR) and their related 95% CI. The congruity between direct and indirect evidence was validated by node splitting studies. If no significant contradiction was identified, a consistency model was employed to examine the relative effects of the interventions. Alternatively, an inconsistency model was utilized. Probabilities were computed to rank each treatment and determine their respective ranks. We employed network diagrams to demonstrate the interconnectedness of treatment measures.

## 3 Results

### 3.1 Search results

**Figure 1:**
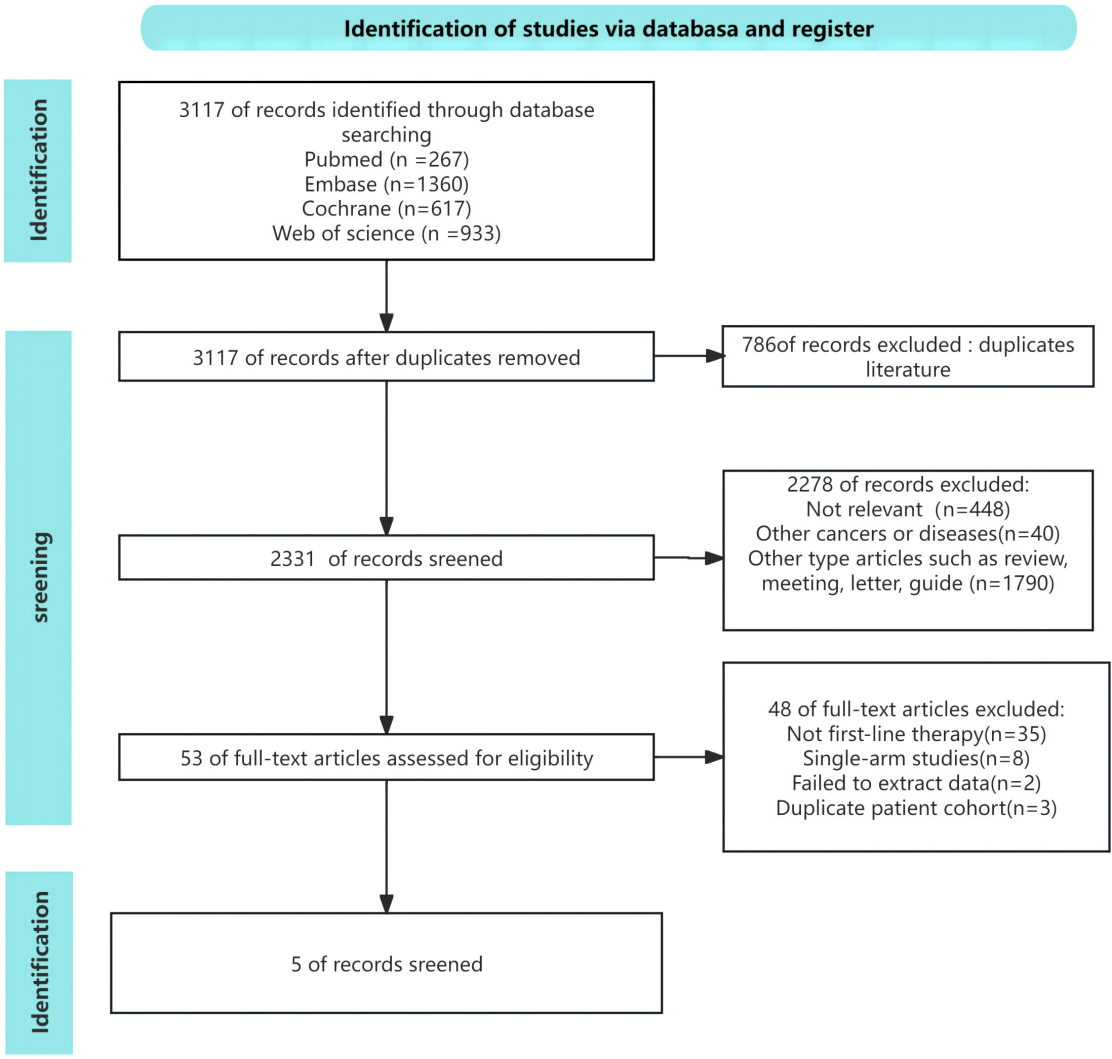
Risk of bias assessment diagram.

Following the initial search, a total of 3,117 article were identified. By eliminating redundant research, the total number of records was reduced to 2331. Out of these, a total of 2,278 papers were eliminated after assessing the titles and abstracts. Following a thorough examination of the entire text, a total of five articles were selected for inclusion in this meta-analysis, including of three three-arm RCTs (12, 13, 19) and two two-arm RCTs (14, 15). **Figure 1** illustrated the comprehensive procedure of selecting and rejecting literature.

### 3.2 Patient characteristics

**Table 1** presented detailed data on the characteristics of included studies and patients. The study comprised a sample of 4749 patients who were diagnosed with metastatic or advanced UC. Patients in the controlled groups in all RCTs received either gemcitabine plus cisplatin or gemcitabine plus carboplatin as their intervention. These trials investigated nine different treatment regimens, which can be categorized into four types: (1) ICI combined with chemotherapy, including of atezolizumab plus chemotherapy (Atezo + Chemo), pembrolizumab plus chemotherapy (Pembro + Chemo), and nivolumab plus chemotherapy (Nivol + Chemo); (2) ICI alone, including of atezolizumab (Atezo), pembrolizumab (Pembro), durvalumab (Durva), and durvalumab plus tremelimumab (Durva + Treme); (3) enfortumab vedotin plus pembrolizumab (EV + Pembro); (4) chemotherapy alone (Chemo). Baseline patient characteristics, such as drug, number of patients, age, gender, performance status, primary tumor site, disease status, PD-L1 status, cisplatin eligibility, follow-up duration, were shown in **Table 1**.

**Table 1:** Characteristics of included studies and patients.

Study	IMvigor130			DANUBE			KEYNOTE361			CheckMate 901		EV-302	
Year	2020			2020			2021			2023		2024	
Clinical Trials.gov	NCT02807636			NCT02516241			NCT02853305			NCT03036098		NCT04223856	
Regimens	Atezolizumab monotherapy vs atezolizumab plus platinum-based chemotherapy vs chemotherapy (gemcitabine plus cisplatin or gemcitabine plus carboplatin)			Durvalumab monotherapy vs durvalumab plus tremelimumab vs chemotherapy (gemcitabine plus cisplatin or gemcitabine plus carboplatin)			Pembrolizumab monotherapy vs pembrolizumab lus platinum-based chemotherapy vs chemotherapy (gemcitabine plus cisplatin or gemcitabine plus carboplatin)			Nivolumab plus gemcitabine-cisplatin vs gemcitabine-cisplatin		Enfortumab Vedotin plus pembrolizumab vs gemcitabine with cisplatin or carboplatin	
Group	Atezo + Chemo	Atezo	Chemo	Durva + Treme	Durva	Chemo	Pembro + Chemo	Pembro	Chemo	Nivol + Chemo	Chemo	EV + Pembro	Chemo
Number	451	362	400	342	346	344	351	307	352	304	304	442	444
Age	69 (62–75)	67 (62–74)	67 (61–73)	68 (60–73)	67 (60–73)	68 (60–73)	69 (41–91)	68 (29–89)	69 (36–90)	65 (32–86)	65 (35–85)	69 (37–87)	69 (22–91)
ECOG PS >1	13%	9%	10%	0%	0%	0%	7%	8%	6%	0.7%	0%	3.4%	2.5%
Disease status (metastatic)	89%	88%	92%	96%	97%	94%	NA	NA	NA	85.9%	88.5%	95.2%	94.6%
High PD-L1	24%	24%	23%	60%	60%	60%	45%	52%	45%	36.5%	36.2%	58.0%	57.9%
Median follow-up (95% CI)	11.8 (6.1–17.2)			41.2 (37.9–43.2)			31.7 (27.7–36.0)			33.6 (7.4 - 62.4)		17.2	
Primary endpoint	PFS、OS			OS			PFS、OS			PFS、OS		PFS、OS	

### 3.3 Risk of Bias

**Figure 2:**
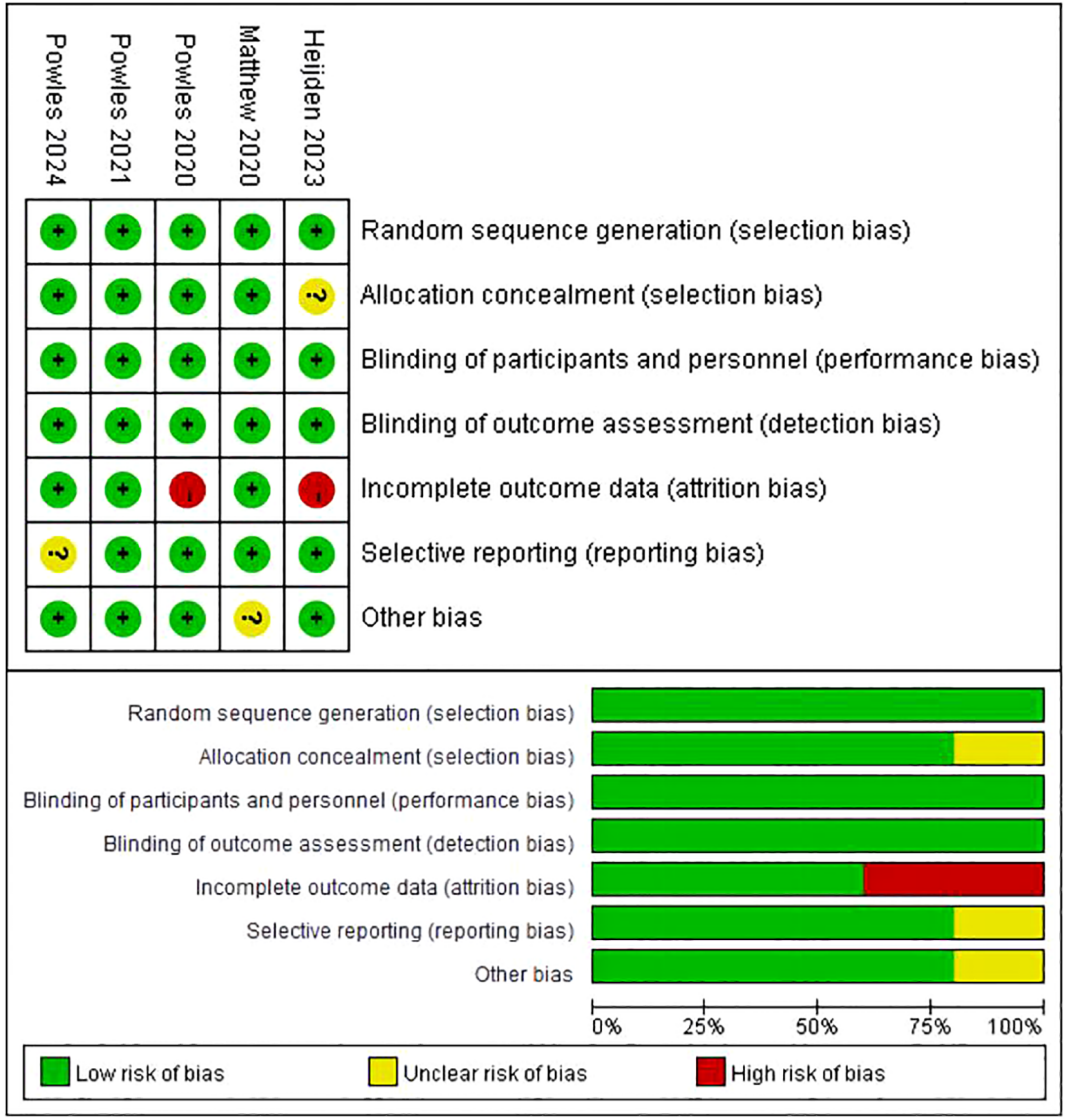
Flow chart of literature search strategies.

The evaluation of the risk of bias is condensed in **Figure 2**. Out of the five studies, all five studies reported an adequate randomized sequence, four studies had appropriate allocation concealment, five studies had clear blinding of participants, five studies had blinding of outcome assessors, three studies had complete outcome data, four studies had no selective reporting, and four studies had no other bias.

### 3.4 Network meta-analysis

#### 3.4.1 OS

**Figure 3:**
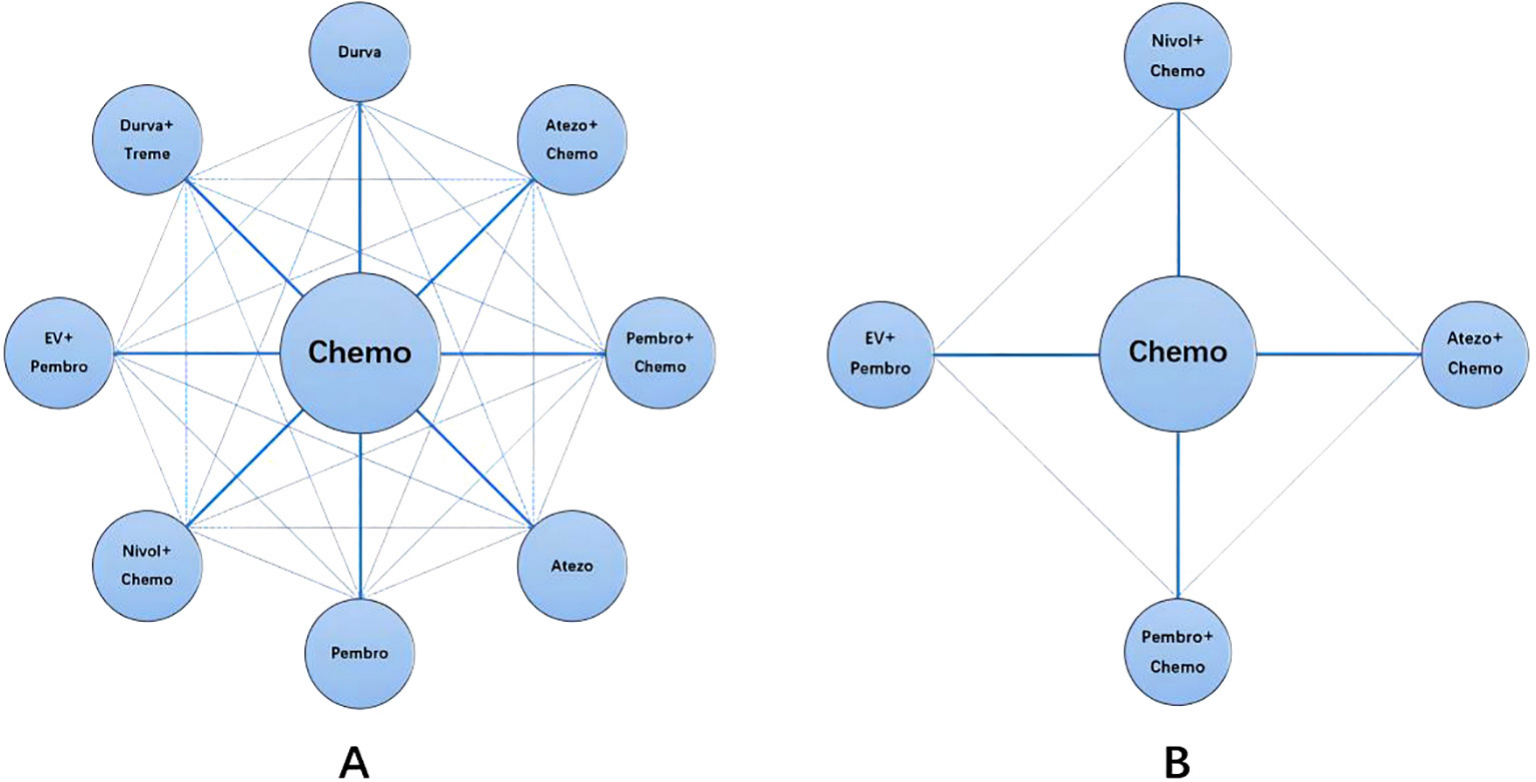
Network diagram. [**(A)** Network diagram for OS, CR, ORR and Grade≥3 AEs; **(B)** Network diagram for PFS].

All five trials provided data on OS, which included nine different treatment regimens (**Figure 3A**). A node-splitting analysis was conducted to assess the inconsistency, and no significant statistical discrepancy was found between the direct and indirect evidence. Thus, we conducted a NMA utilizing a consistency model. **Table 2** displayed the pooled HR obtained from the network meta-analysis, which examined the relationship between regimen and OS in patients with advanced or metastatic UC. The findings demonstrated that EV + Pembro exhibited a substantial advantage in terms of OS when compared to Atezo, Atezo + Chemo, Chemo, Durva, Durva + Treme, Nivol + Chemo, Pembro, and Pembro + Chemo. There were no statistically significant differences observed among the treatment groups, including Atezo, Atezo + Chemo, Chemo, Durva, Durva + Treme, Nivol + Chemo, Pembro, and Pembro + Chemo.

**Table 2:** Pooled HR derived from the network meta-analysis examining the relationship between regimen and OS in patients with advanced or metastatic UC.

Atezo								
1.09 (0.97 - 1.23)	Atezo + Chemo							
1.01 (0.92 - 1.10)	0.92 (0.85 - 1.00)	Chemo						
1.06 (0.93 - 1.21)	0.97 (0.86 - 1.10)	1.05 (0.95 - 1.16)	Durva					
1.08 (0.96 - 1.21)	0.99 (0.89 - 1.11)	1.07 (0.99 - 1.16)	1.02 (0.90 - 1.15)	Durva + Treme				
1.40 (1.23 - 1.59)	1.28 (1.13 - 1.45)	1.39 (1.27 - 1.52)	1.32 (1.15 - 1.51)	1.29 (1.15 - 1.46)	EV + Pembro			
1.12 (0.99 - 1.27)	1.03 (0.91 - 1.16)	1.11 (1.02 - 1.22)	1.06 (0.93 - 1.21)	1.04 (0.92 - 1.17)	0.80 (0.70 - 0.91)	Nivol + Chemo		
1.05 (0.93 - 1.18)	0.96 (0.85 - 1.07)	1.04 (0.96 - 1.12)	0.99 (0.87 - 1.12)	0.97 (0.87 - 1.08)	0.75 (0.66 - 0.84)	0.93 (0.82 - 1.05)	Pembro	
1.08 (0.96 - 1.21)	0.98 (0.88 - 1.10)	1.07 (0.99 - 1.15)	1.02 (0.90 - 1.15)	0.99 (0.89 - 1.11)	0.77 (0.68 - 0.87)	0.96 (0.85 - 1.08)	1.03 (0.92 - 1.15)	Pembro + Chemo

Pooled HR (95% credible interval) derived from network meta-analysis.

Colored indicates a statistically significant comparison.

**Figure 4:**
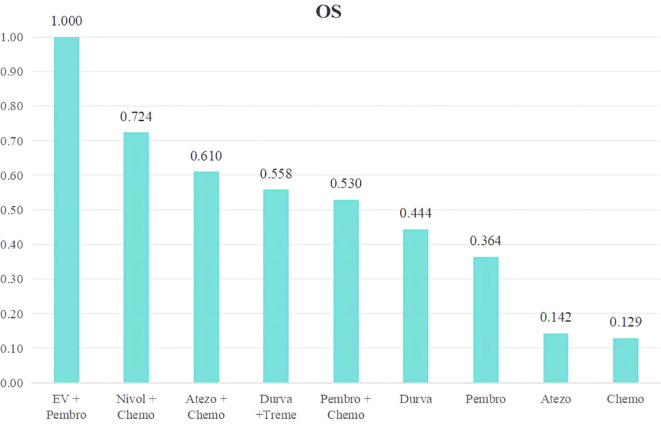
Rank bar charts for OS.

We conducted a ranking of the therapeutic effectiveness of the 9 treatment regimens for advanced or metastatic UC. We then presented this ranking using bar charts, where taller bars represent higher OS rates and better treatment effectiveness (**Figure 4**). The top five therapy regimens, ranked by efficacy, were EV + Pembro (1.000), Nivol + Chemo (0.724), Atezo + Chemo (0.610), Durva + Treme (0.558), and Pembro + Chemo (0.530).

A subgroup analysis in patients with high PD-L1 expression was performed (**Supplementary Material 2**). The subgroup analysis revealed that treatment regimens involving EV + Pembro exhibited a substantial advantage in terms of OS when compared to Chemo, Durva, Durva + Treme, Pembro, and Pembro + Chemo, but not Atezo, Atezo + Chemo or Nivol + Chemo.

#### 3.4.2 PFS

A total of 4 studies reported PFS, involving five treatment regimens (**Figure 3B**). **Table 3** presented the combined HR derived from the network meta-analysis, which investigated the correlation between treatment regimen and PFS in individuals with metastatic UC. The findings demonstrated that the combination of EV and Pembro treatment regimens had a notable advantage in terms of PFS when compared to four alternative treatment regimens. In addition, the combinations of Nivol + Chemo, Pembro + Chemo, and Atezo + Chemo demonstrated a notable advantage in terms of PFS when compared to Chemo alone.

**Table 3:** Pooled HR derived from the network meta-analysis examining the relationship between regimen and PFS in patients with advanced or metastatic UC.

Atezo + Chemo				
0.92 (0.86 -0.98)	Chemo			
1.30 (1.17 -1.44)	1.41 (1.31 -1.53)	EV + Pembro		
1.06 (0.95 -1.18)	1.15 (1.06 -1.26)	0.82 (0.73 -0.92)	Nivol + Chemo	
1.02 (0.92 -1.13)	1.11 (1.03 -1.20)	0.79 (0.71 -0.88)	0.97 (0.86 -1.09)	Pembro + Chemo

Pooled HR (95% credible interval) derived from network meta-analysis.

Colored indicates a statistically significant comparison.

**Figure 5:**
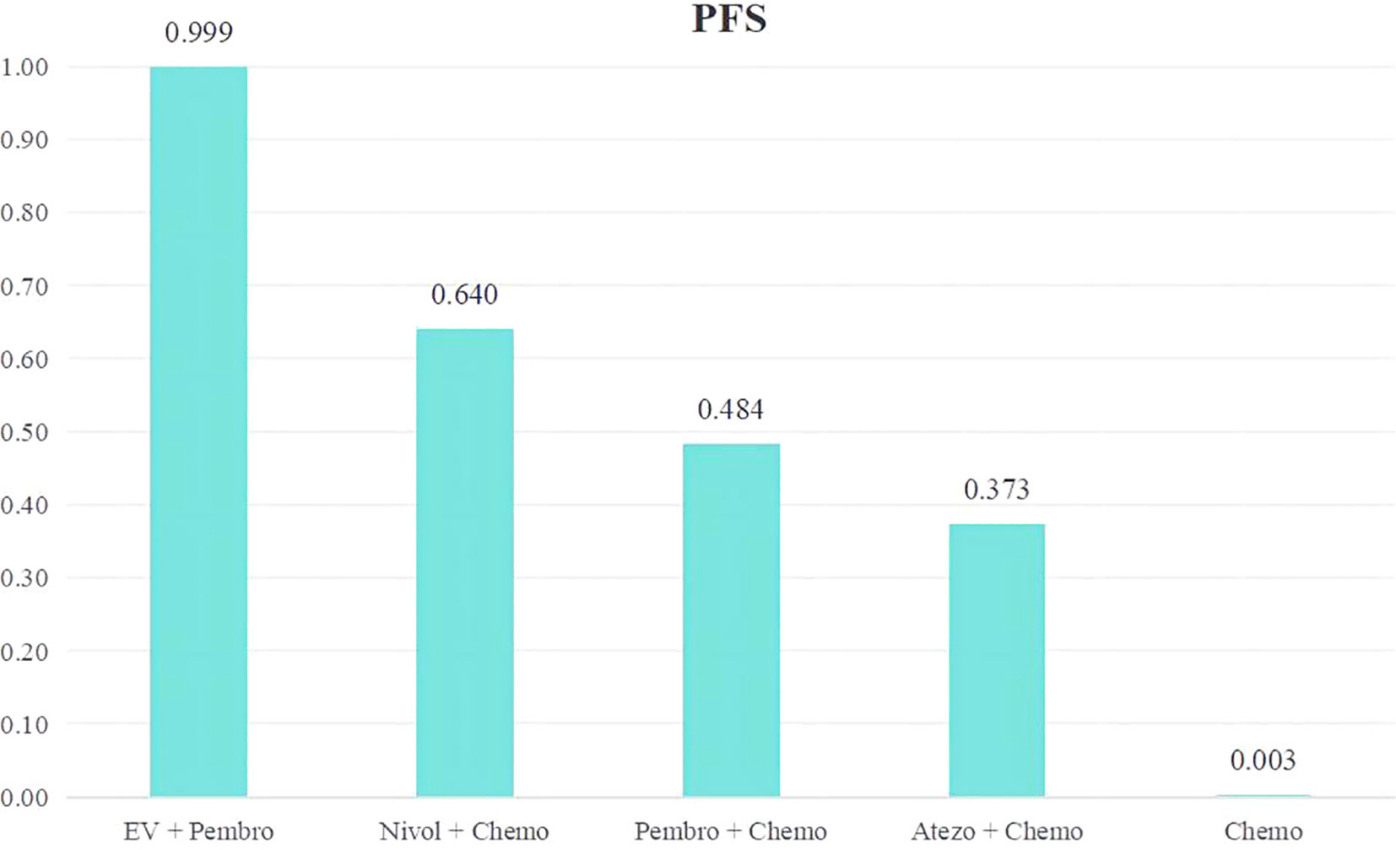
Rank bar charts for PFS.

A ranking of the therapeutic effectiveness of the five treatment regimens for advanced or metastatic UC was conducted (**Figure 5**). The top five therapy regimens, ranked by efficacy, were EV + Pembro (0.999), Nivol + Chemo (0.640), Pembro + Chemo (0.484), Atezo + Chemo (0.373) and Chemo (0.003).

#### 3.4.3 CR

A total of 5 studies reported CR, involving 9 treatment regimens (**Figure 3A**). **Table 4** displayed the pooled OR obtained from a network meta-analysis examining the relationship between treatment and CR in metastatic UC. The majority of the pairwise comparisons among the nine regimens exhibited significant statistical differences. Compared with Chemo, the EV + Pembro, Nivol + Chemo and Atezo + Chemo regimens had a significantly better CR.

**Table 4:** Pooled OR derived from the network meta-analysis examining the relationship between regimen and CR in patients with advanced or metastatic UC.

EV + Pembro								
1.39 (0.79 - 2.45)	Nivol + Chemo							
1.46 (0.81 - 2.65)	1.05 (0.55 - 2.02)	Atezo + Chemo						
2.25 (1.29 - 3.92)	1.62 (0.87 - 3.00)	1.54 (0.80 - 2.93)	Pembro + Chemo					
2.29 (1.16 - 4.53)	1.65 (0.79 - 3.42)	1.56 (0.73 - 3.33)	1.02 (0.49 - 2.11)	Durva + Treme				
2.32 (1.18 - 4.58)	1.67 (0.80 - 3.47)	1.58 (0.74 - 3.38)	1.03 (0.50 - 2.13)	1.01 (0.44 - 2.31)	Durva			
2.88 (2.03 - 4.08)	2.06 (1.33 - 3.21)	1.96 (1.21 - 3.17)	1.28 (0.83 - 1.97)	1.25 (0.70 - 2.25)	1.24 (0.69 - 2.22)	Chemo		
3.21 (1.63 - 6.34)	2.31 (1.11 - 4.79)	2.19 (1.03 - 4.67)	1.43 (0.69 - 2.95)	1.40 (0.61 - 3.20)	1.38 (0.61 - 3.16)	1.12 (0.62 - 2.00)	Atezo	
3.21 (1.78 - 5.81)	2.31 (1.20 - 4.42)	2.19 (1.11 - 4.32)	1.43 (0.75 - 2.72)	1.40 (0.66 - 2.98)	1.38 (0.65 - 2.94)	1.12 (0.69 - 1.80)	1.00 (0.47 - 2.12)	Pembro

Pooled OR (95% credible interval) derived from network meta-analysis.

Colored indicates a statistically significant comparison.

**Figure 6:**
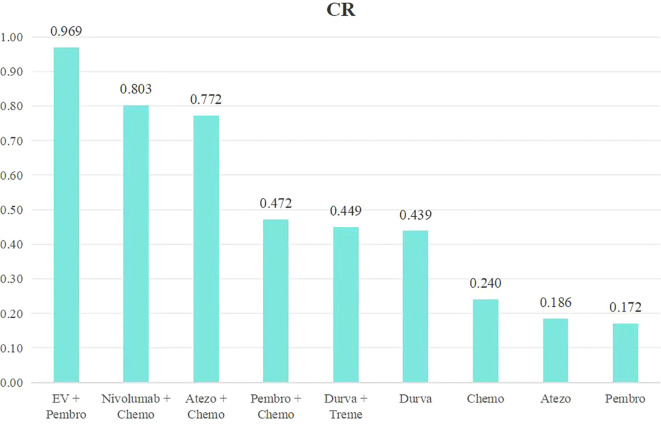
Rank bar charts for CR.

An analysis was conducted to rank the therapeutic efficiency of nine treatment regimens for metastatic UC (**Figure 6**). The top five therapy regimens, ranked by efficacy, were EV + Pembro (0.969), Nivol + Chemo (0.803), Atezo + Chemo (0.772), Pembro + Chemo (0.472), Durva + Treme (0.449) (**Figure 4D**).

#### 3.4.4 ORR

A total of 5 studies reported ORR, involving 9 treatment regimens (**Figure 3A**). **Table 5** displayed the combined OR obtained from a network meta-analysis examining the relationship between treatment and ORR in metastatic UC. The majority of the pairwise comparisons among the nine regimens exhibited significant statistical differences.

**Table 5:** Pooled OR derived from the network meta-analysis examining the relationship between regimen and ORR in patients with advanced or metastatic UC.

EV + Pembro								
1.46 (0.96 - 2.24)	Nivol + Chemo							
1.77 (1.18 - 2.65)	1.21 (0.78 - 1.87)	Pembro + Chemo						
2.27 (1.54 - 3.34)	1.55 (1.02 - 2.36)	1.28 (0.86 - 1.92)	Atezo + Chemo					
2.62 (1.99 - 3.45)	1.79 (1.30 - 2.47)	1.48 (1.10 - 2.00)	1.16 (0.88 - 1.52)	Chemo				
4.46 (2.95 - 6.72)	3.04 (1.95 - 4.74)	2.52 (1.64 - 3.85)	1.96 (1.30 - 2.95)	1.70 (1.25 - 2.30)	Durva + Treme			
4.92 (3.22 - 7.51)	3.36 (2.13 - 5.29)	2.78 (1.79 - 4.30)	2.17 (1.42 - 3.30)	1.87 (1.36 - 2.58)	1.10 (0.71 - 1.72)	Pembro		
6.92 (4.55 - 10.52)	4.72 (3.01 - 7.41)	3.91 (2.53 - 6.03)	3.05 (2.01 - 4.62)	2.64 (1.92 - 3.62)	1.55 (1.00 - 2.41)	1.41 (0.90 - 2.21)	Atezo	
7.32 (4.80 - 11.16)	5.00 (3.17 - 7.87)	4.13 (2.67 - 6.40)	3.22 (2.12 - 4.91)	2.79 (2.02 - 3.84)	1.64 (1.05 - 2.56)	1.49 (0.94 - 2.34)	1.06 (0.67 - 1.66)	Durva

Pooled OR (95% credible interval) derived from network meta-analysis.

Colored indicates a statistically significant comparison.

**Figure 7:**
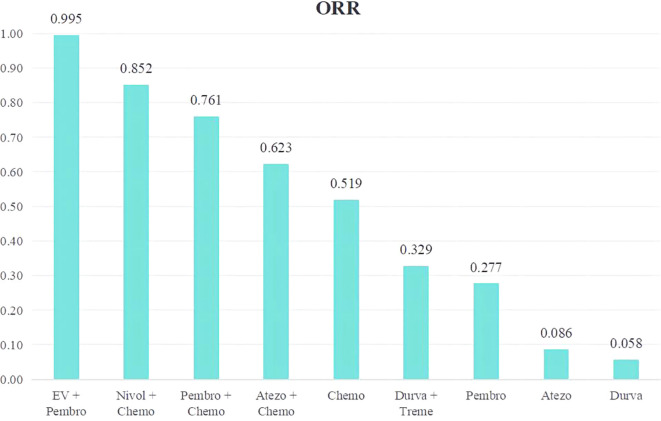
Rank bar charts for ORR.

An analysis was done to rank the therapeutic efficiency of nine treatment regimens for metastatic UC (**Figure 7**). The top five therapy regimens, ranked by efficacy, were EV + Pembro (0.995), Nivol + Chemo (0.852), Pembro + Chemo (0.761), Atezo + Chemo (0.623), and Chemo (0.519).

#### 3.4.5 Grade≥3 AEs

A total of 5 studies reported Grade≥3 AEs, involving 9 treatment regimens (**Figure 3A**). **Table 6** displayed pooled OR derived from a network meta-analysis examining the association of regimens with Grade≥3 AEs in metastatic UC. Significant statistical differences were seen in the majority of pairwise comparisons among the nine regimens.

**Table 6:** Pooled OR derived from the network meta-analysis examining the relationship between regimen and Grade ≥ 3 AEs in patients with advanced or metastatic UC.

Atezo								
0.93 (0.53- 1.61)	Durva + Treme							
0.33 (0.20- 0.56)	0.36 (0.22- 0.60)	Durva						
0.28 (0.17- 0.48)	0.30 (0.18- 0.51)	0.84 (0.52- 1.36)	Pembro					
0.18 (0.11- 0.29)	0.20 (0.12- 0.31)	0.54 (0.35- 0.83)	0.64 (0.41- 1.00)	EV + Pembro				
0.10 (0.07- 0.15)	0.11 (0.07- 0.16)	0.30 (0.22- 0.42)	0.36 (0.25- 0.51)	0.56 (0.42- 0.73)	Chemo			
0.10 (0.05- 0.18)	0.11 (0.06- 0.19)	0.29 (0.17- 0.52)	0.35 (0.20- 0.63)	0.54 (0.32- 0.94)	0.98 (0.61- 1.57)	Atezo + Chemo		
0.07 (0.04- 0.12)	0.07 (0.04- 0.12)	0.20 (0.12- 0.33)	0.23 (0.14- 0.40)	0.37 (0.23- 0.59)	0.66 (0.44- 0.97)	0.67 (0.36- 1.24)	Pembro + Chemo	
0.06 (0.04- 0.10)	0.06 (0.04- 0.11)	0.18 (0.11- 0.28)	0.21 (0.13- 0.34)	0.33 (0.22- 0.50)	0.59 (0.43- 0.82)	0.61 (0.34- 1.07)	0.90 (0.54- 1.50)	Nivol + Chemo

Pooled OR (95% credible interval) derived from network meta-analysis.

Colored indicates a statistically significant comparison.

**Figure 8:**
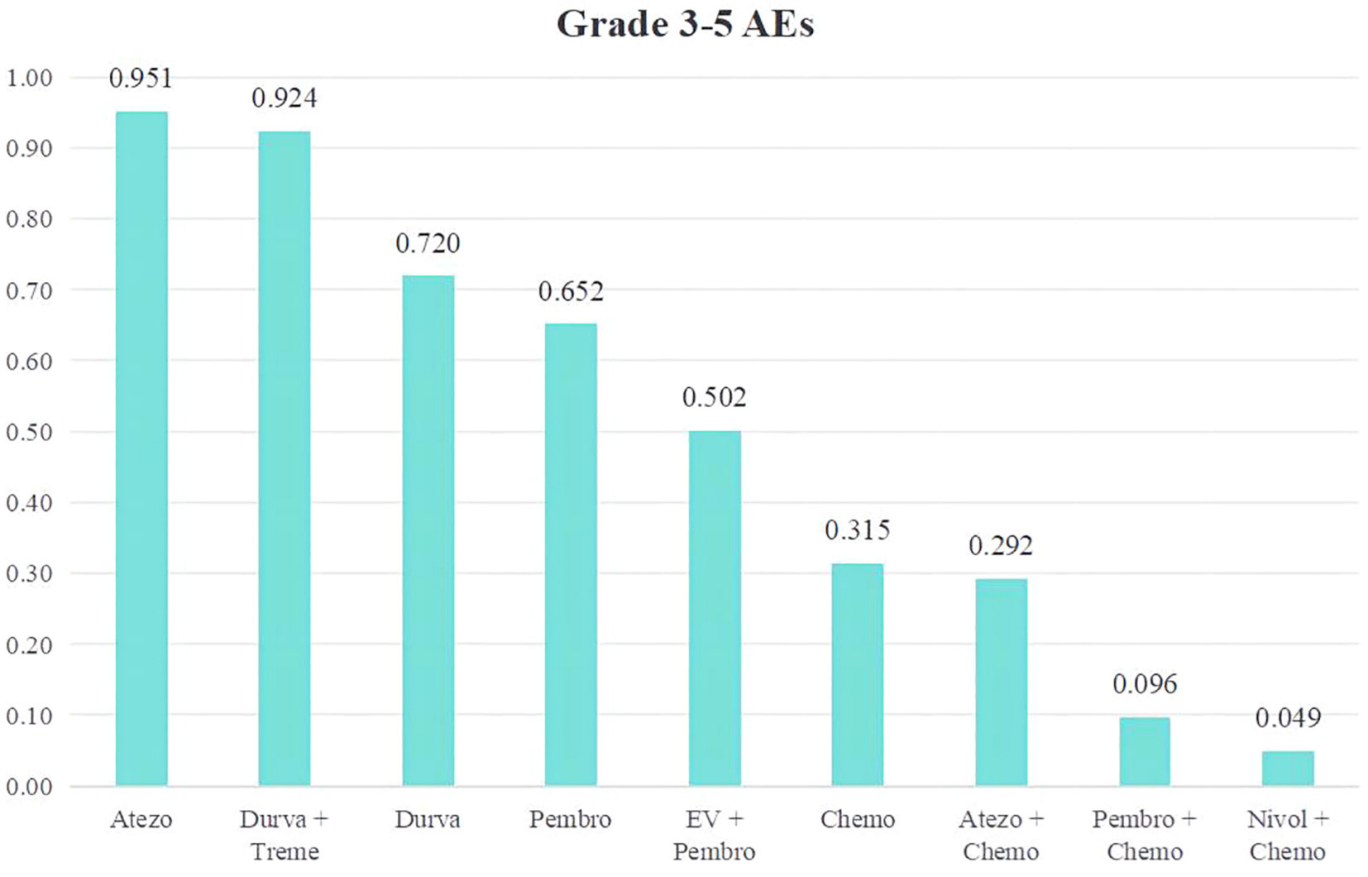
Rank bar charts for Grade ≥ 3 AEs.

We conducted a ranking of the Grade≥3 AEs rate of the 9 treatment regimens for advanced or metastatic UC. A ranking was presented using bar charts, where higher bars indicating greater toxicity due to the regimen (**Figure 8**).The top five therapy regimens, ranked by safety, were Atezo (0.049), Durva + Treme (0.076), Durva (0.280), Pembro (0.348), EV + Pembro (0.492).

### 3.5 Publication bias

**Figure 9:**
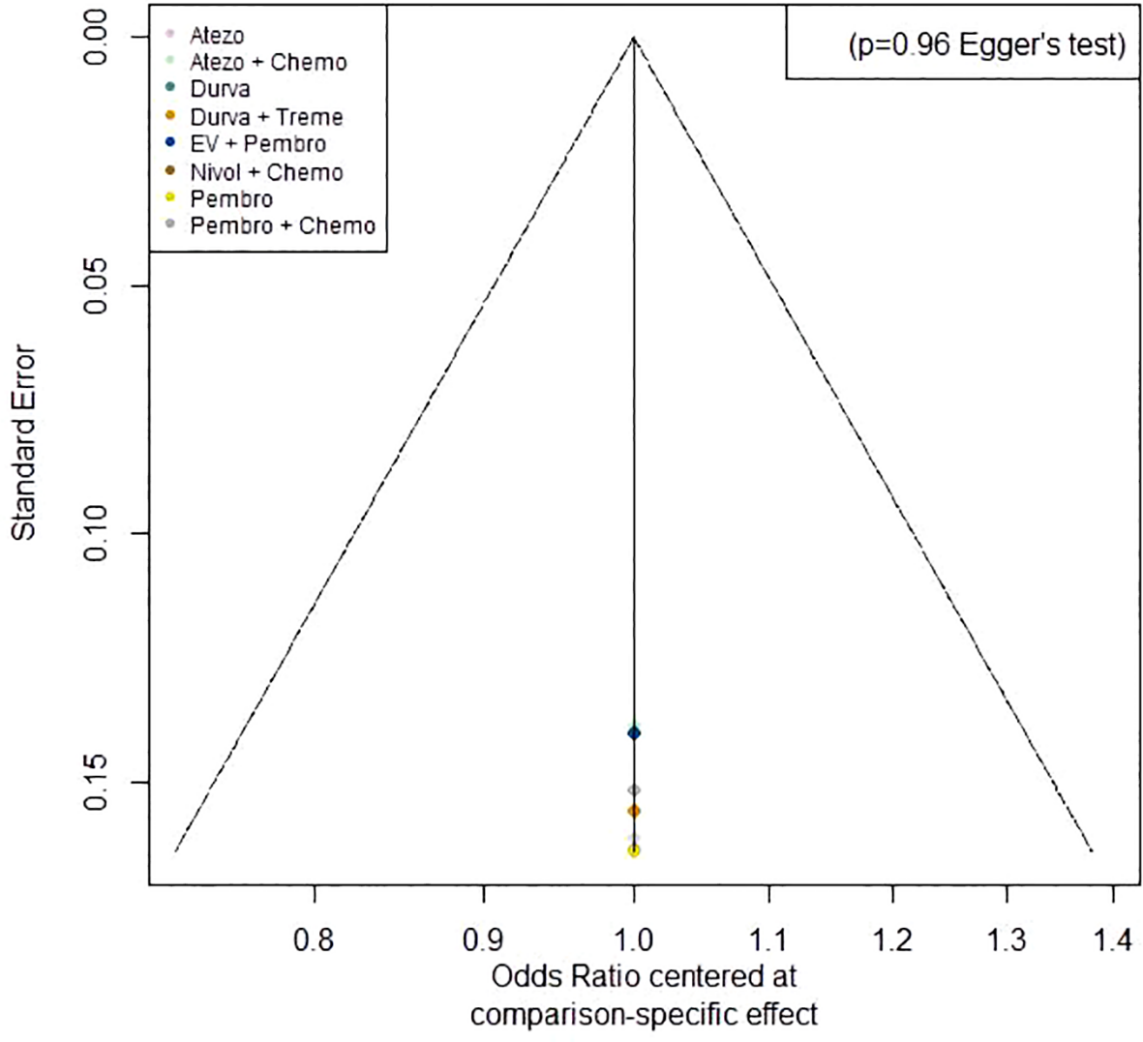
Funnel plot on ORR.

A funnel plot on ORR was generated using Egger’s test method. The plot revealed that the majority of the study’s data points were concentrated around the center line, with only a few points scattered on either side. The p-value of 0.96 indicates a low probability of publication bias (**Figure 9**).

## 4 Discussion

Due to the notable therapeutic advantages observed in patients with advanced or metastatic UC, there has been a rise in the number of clinical trials aimed at assessing the safety and effectiveness of first-line immunotherapy-containing regimens compared with chemotherapy in advanced or metastatic UC. This study conducted a network meta-analysis to evaluate the safety and efficacy of first-line immunotherapy in patients with advanced or metastatic UC.

### 4.1 Role of ICI plus chemotherapy

Chemoimmunotherapy is shown to have a more favorable OS and PFS compared to chemotherapy alone for malignancies in the advanced or metastatic stage in several tumor types (20–23). Our study included three chemoimmunotherapy regimens: Atezo + Chemo, Pembro + Chemo and Nivol + Chemo. All the pairwise comparisons among four chemoimmunotherapy regimens exhibited no significant statistical difference in terms of OS, PFS, CR and ORR. The results of our study demonstrated that the combination of immunotherapy and chemotherapy as first-line treatment led to more favorable clinical outcomes in comparison to chemotherapy alone for advanced or metastatic UC who are eligible for platinum-based chemotherapy. This was apparent in relation to PFS, CR and ORR. However, despite the promising trend in chemoimmunotherapy, there was no statistically significant difference in OS between chemoimmunotherapy and chemotherapy. The results provide evidence for the efficacy of immunotherapy in combination with platinum-based chemotherapy as a potential first-line treatment option for advanced or metastatic UC.

The reported inconsistencies in phase 3 trials may be partly attributed to potential variations in the immunomodulatory impacts of cisplatin and carboplatin (24–26). Upon analyzing the IMvigor130 study, it was found that pretreatment tumors with higher levels of PD-L1 expression were linked to better outcomes in patients who received gemcitabine plus cisplatin, but not in those who received gemcitabine plus carboplatin (25). Analysis of circulating immune cells using single-cell RNA sequencing showed that treatment with gemcitabine plus cisplatin, but not gemcitabine plus carboplatin, resulted in an increase in the activity of immune-related transcriptional programs, specifically those involved in antigen presentation (25). These observations provide more evidence of the possible immune-stimulating effects of cisplatin and suggest that using cisplatin-based chemotherapy, rather than carboplatin, may be especially beneficial when combined with ICI for treating metastatic UC.

### 4.2 Role of ICI alone

The study encompassed four ICI alone regimens, namely Atezo, Durva, Pembro, and Durva + Treme. The findings of our study indicate that the use of ICI alone as the initial treatment resulted in a reduced ORR compared to chemotherapy alone for advanced or metastatic UC. Furthermore, there was no statistically significant difference in OS between ICI alone and chemotherapy.

Over the course of multiple years, the OS results for individuals with metastatic UC had reached a point where there was no further improvement when treated with chemotherapy. Pembrolizumab and other ICIs have been established as the standard treatment for platinum refractory patients, as demonstrated by randomized trials (27). Recently, atezolizumab and pembrolizumab were authorized as initial therapies for patients with metastatic UC who are unable to receive cisplatin and whose tumors have a high expression of PD-L1 (28, 29). Moreover, our study revealed that incorporating tremelimumab can potentially augment both the ORR and the side effects of durvalumab in patients with previously untreated metastatic UC, but it did not have the ability to boost OS. Further research is required to examine the efficacy of CTLA-4 inhibitors in the treatment of metastatic UC, specifically in patients with positive PD-L1 biomarker (19). Simultaneously, the development of clinical prediction models and the exploration of more precise predictive biomarkers can enhance the ability to forecast the effectiveness of ICI (30).

### 4.3 Role of EV + Pembro

The findings of our study demonstrated that the combination of EV + Pembro yielded the most favorable clinical outcomes compared to the other eight treatment regimens. This was evident in terms of OS, PFS, CR and ORR. Besides, a decreased incidence of treatment-related adverse events of grade 3 or higher was seen compared to the chemotherapy group.

Nectin-4 is a protein that is found in higher levels in UC and other types of cancer. It has become an attractive focus for new treatments that target tumors, especially when used in antibody-drug conjugates (ADCs), which are a type of anti-cancer therapy that is gaining popularity (31). Enfortumab vedotin, a targeted therapy that binds to nectin-4, and pembrolizumab, an immunotherapy that inhibits the PD-1 protein, have separately shown to improve survival in patients with advanced or metastatic UC who have already received treatment (13, 32–35). Preclinical investigations shown that the combination of enfortumab vedotin and a PD-1 inhibitor exhibited heightened anticancer efficacy and resulted in long-lasting antitumor immunity (36). These data indicate that the two drugs work together in a way that complements each other’s mechanisms of action. The findings of our study demonstrated that the combination of EV and Pembro resulted in superior clinical outcomes and safety when compared to platinum-based chemotherapy. It is worth noting that the EV-302 trial did not involve any preselection based on cisplatin eligibility status or biomarkers, such as nectin-4 and programmed death ligand 1 (PD-L1) expression (15). These findings indicate that the combination of EV and Pembro could be a viable first-line treatment choice for patients with advanced or metastatic UC. This treatment option resulted in considerably improved outcomes and safety compared to chemotherapy, regardless of the patient’s eligibility for cisplatin or their biomarker status. Based on the results of phase 3 EV-302 trial, which showed that the combination of enfortumab vedotin and pembrolizumab produced high incidences of response and durable responses, the United States has expedited the approval of this medication for all patients with metastatic UC (37, 38). The findings of our meta-analysis reinforced this approval.

The subgroup analysis regarding high PD-L1 expression revealed that EV + Pembro exhibited a substantial advantage in terms of OS when compared to Chemo, Durva, Durva + Treme, Pembro, and Pembro + Chemo, but not Atezo, Atezo + Chemo or Nivol + Chemo. This result suggested that in patients with high PD1 expression, EV + Pembro was the best option regarding OS. Besides, Atezo, Atezo + Chemo and Nivol + Chemo were also alternative treatments.

The current frontline standard of care regimens for metastatic UC in the US are pembro + EV or platinum chemo + nivolumab based on the phase 3 trials where each regimen was compared to chemo alone. The findings of this meta-analysis revealed that pembro + EV has an advantage over Nivol + Chemo regarding of OS, PFS, ORR and Grade ≥ 3 AEs. It is important to emphasize the comparison of these 2 regimens in this meta-analysis, as likely there will never be a randomized trial of pembro+EV vs Nivol + Chemo. This is the most clinically relevant and impactful comparison from the presented analysis.

It is necessary to further discuss the significance of the variant histologies of UC. Moschini M et al. discovered that patients with the small cell variety exhibited a detrimental impact on survival following radical cystectomy (RC) (39). Claps F et al. reported that over 25% of patients exhibited variable histologies (VHs) at the time of RC. In comparison to pure UC, clear-cell, plasmacytoid, small-cell, and sarcomatoid variant histologies were linked to poorer disease-specific survival (DSS), whereas lymphoepithelioma-like variant histology exhibited a DSS advantage (40). In comparison to patients diagnosed with conventional UC at the same disease stage, survival rates did not seem to be markedly inferior across the reports. However, when evaluated by individual subtype, certain types, including micropapillary, plasmacytoid, small-cell, and sarcomatoid, were found to be independently linked to negative survival outcomes, while others were not (41, 42). Wood AM et al. reported that percent Micropapillary variant UC component on transurethral resection is correlated with a heightened risk of undetected lymph node metastases at RC (43). Unfortunately, due to the data limitation, we failed to performed a subgroup meta-analysis regarding variant histologies of UC.

### 4.4 Strength and limitation

The strength of our study was clear. This study is the first network meta-analysis to evaluate the efficacy and safety of first-line immunotherapy-containing regimens compared with chemotherapy for patients with advanced or metastatic UC. All the studies considered in the analysis were high-quality RCTs. Different treatment regimens included in the study were ranked statistically and intuitively using NMA. The assessment of each regimen was conducted completely by considering both efficacy and safety occurrences. Without a doubt, our study possesses specific constraints. First, in order to complete the NMA, single-arm trials were not considered, and the sample size was rather small. Furthermore, the five trials showed significant variation in terms of the characteristics of the patients included and the treatment plans used. ECOG PS,PD-L1 expression, variant histologies and age of patient might be potential sources of heterogeneity. However, due to the data limitation, we failed to performed subgroup meta-analyses regarding these factors except for PD-L1 expression. Therefore, it is crucial to show prudence when interpreting our findings.

## 5 Conclusion

Our findings demonstrated that the combination of EV + Pembro as the initial treatment option led to significantly enhanced efficacy and safety in comparison to chemotherapy for advanced or metastatic UC. The combination of ICI and chemotherapy as the initial treatment led to a longer PFS and a higher ORR compared to chemotherapy alone. However, it did not result in a longer OS. Additionally, the combination treatment was associated with increased toxicity. ICI alone as the initial treatment option demonstrated comparable OS and reduced toxicity in comparison to chemotherapy, while it resulted in a decreased ORR.

## Data Availability

The datasets presented in this study can be found in online repositories. The names of the repository/repositories and accession number(s) can be found in the article/**Supplementary Material**.
