# Supplementary material for: Efficacy and safety of first-line immunotherapy-containing regimens compared with chemotherapy for advanced or metastatic urothelial carcinoma: a network meta-analysis of randomized controlled trials
Source: Front Oncol. 2024 Dec 11;14:1453338. doi: 10.3389/fonc.2024.1453338 (PMC11668658; doi:10.3389/fonc.2024.1453338)
Supplement: Supplementary file 1 [file Table1.docx]

**Supplement material 1. The searching record in detail**

| PubMed | | |
| --- | --- | --- |
| No. | Query | Results |
| 1 | ((((((((((((((((((((Urinary Bladder Neoplasms[MeSH Terms]) OR (Neoplasm, Urinary Bladder[Title/Abstract])) OR (Urinary Bladder Neoplasm[Title/Abstract])) OR (Bladder Tumors[Title/Abstract])) OR (Bladder Tumor[Title/Abstract])) OR (Tumor, Bladder[Title/Abstract])) OR (Tumors, Bladder[Title/Abstract])) OR (Neoplasms, Bladder[Title/Abstract])) OR (Bladder Neoplasms[Title/Abstract])) OR (Bladder Neoplasm[Title/Abstract])) OR (Neoplasm, Bladder[Title/Abstract])) OR (Urinary Bladder Cancer[Title/Abstract])) OR (Cancer, Urinary Bladder[Title/Abstract])) OR (Malignant Tumor of Urinary Bladder[Title/Abstract])) OR (Cancer of the Bladder[Title/Abstract])) OR (Bladder Cancer[Title/Abstract])) OR (Bladder Cancers[Title/Abstract])) OR (Cancer, Bladder[Title/Abstract])) OR (Cancer of Bladder[Title/Abstract])) OR (urothelial carcinoma[Title/Abstract] OR bladder cancer[Title/Abstract])) OR (urothelial cancer[Title/Abstract]) | 85099 |
| 2 | immunotherapy[Title/Abstract] OR immune checkpoint inhibitor[Title/Abstract] OR PD-1 inhibitor[Title/Abstract] OR PD-L1 inhibitor[Title/Abstract] OR Atezolizumab[Title/Abstract] OR Nivolumab[Title/Abstract] OR Durvalumab[Title/Abstract] OR Pembrolizumab[Title/Abstract] OR Avelumab[Title/Abstract] OR Erdafitinib[Title/Abstract] OR Enfortumab Vedotin[Title/Abstract] OR Sacituzumab Govitecan[Title/Abstract] OR Nectin-4[Title/Abstract] OR disitamab vedotin[Title/Abstract] OR FGFR[Title/Abstract] | 161306 |
| 3 | first-line OR untreated OR initial OR primary OR newly-diagnosed OR newly[MeSH Terms] | 4087430 |
| 4 | (randomized controlled trial [pt] OR controlled clinical trial [pt] OR randomized [tiab] OR placebo [tiab] OR clinical trials as topic [mesh:noexp] OR randomly [tiab] OR trial [ti]) NOT (animals [mh] NOT humans [mh]) | 1481889 |
| 5 | 1 AND 2 AND 3 AND 4 | 267 |

| Web of science | | |
| --- | --- | --- |
| No. | Query | Results |
| 1 | ((((((((((((((((((((TS=(Urinary Bladder Neoplasms)) OR TS=(Neoplasm, Urinary Bladder)) OR TS=(Urinary Bladder Neoplasm)) OR TS=(Bladder Tumors)) OR TS=(Bladder Tumor)) OR TS=(Tumor, Bladder)) OR TS=(Tumors, Bladder)) OR TS=(Neoplasms, Bladder)) OR TS=(Bladder Neoplasms)) OR TS=(Bladder Neoplasm)) OR TS=(Neoplasm, Bladder)) OR TS=(Urinary Bladder Cancer)) OR TS=(Cancer, Urinary Bladder)) OR TS=(Malignant Tumor of Urinary Bladder)) OR TS=(Cancer of the Bladder)) OR TS=(Bladder Cancer)) OR TS=(Bladder Cancers)) OR TS=(Cancer, Bladder)) OR TS=(Cancer of Bladder)) OR TS=(urothelial carcinoma)) OR TS=(urothelial cancer) | 106968 |
| 2 | TS=(immunotherapy OR immune checkpoint inhibitor OR PD-1 inhibitor OR PD-L1 inhibitor OR Atezolizumab OR Nivolumab OR Durvalumab OR Pembrolizumab OR Avelumab OR Erdafitinib OR Enfortumab Vedotin OR Sacituzumab Govitecan OR Nectin-4 OR disitamab vedotin OR FGFR) | 234547 |
| 3 | TS=(first-line OR untreated OR initial OR primary OR newly-diagnosed OR newly) | 5004562 |
| 4 | TS=(randomized controlled trial OR controlled clinical trial OR Randomized OR placebo OR clinical trials as topic OR randomly OR Trial OR Prospective) | 3330747 |
| 5 | 1 AND 2 AND 3 AND 4 | 933 |

| Embase | | |
| --- | --- | --- |
| No. | Query | Results |
| 1 | 'urinary bladder neoplasms'/exp OR 'urinary bladder neoplasms' OR (urinary AND ('bladder'/exp OR bladder) AND ('neoplasms'/exp OR neoplasms)) OR 'neoplasm, urinary bladder':ti,ab,kw OR 'urinary bladder neoplasm':ti,ab,kw OR 'bladder tumors':ti,ab,kw OR 'bladder tumor':ti,ab,kw OR 'tumor, bladder':ti,ab,kw OR 'tumors, bladder':ti,ab,kw OR 'neoplasms, bladder':ti,ab,kw OR 'bladder neoplasms':ti,ab,kw OR 'bladder neoplasm':ti,ab,kw OR 'neoplasm, bladder':ti,ab,kw OR 'urinary bladder cancer':ti,ab,kw OR 'cancer, urinary bladder':ti,ab,kw OR 'malignant tumor of urinary bladder':ti,ab,kw OR 'cancer of the bladder':ti,ab,kw OR 'bladder cancer':ti,ab,kw OR 'bladder cancers':ti,ab,kw OR 'cancer, bladder':ti,ab,kw OR 'cancer of bladder':ti,ab,kw OR 'urothelial carcinoma':ti,ab,kw OR 'urothelial cancer':ti,ab,kw | 152941 |
| 2 | immunotherapy:ti,ab,kw OR 'immune checkpoint inhibitor':ti,ab,kw OR 'pd-1 inhibitor':ti,ab,kw OR 'pd-l1 inhibitor':ti,ab,kw OR atezolizumab:ti,ab,kw OR nivolumab:ti,ab,kw OR durvalumab:ti,ab,kw OR pembrolizumab:ti,ab,kw OR avelumab:ti,ab,kw OR erdafitinib:ti,ab,kw OR 'enfortumab vedotin':ti,ab,kw OR 'sacituzumab govitecan':ti,ab,kw OR 'nectin 4':ti,ab,kw OR 'disitamab vedotin':ti,ab,kw OR fgfr:ti,ab,kw | 257265 |
| 3 | 'first line':ti,ab,kw OR untreated:ti,ab,kw OR initial:ti,ab,kw OR primary:ti,ab,kw OR 'newly diagnosed':ti,ab,kw OR newly:ti,ab,kw | 4749593 |
| 4 | 'randomized controlled trial':ab,ti OR 'controlled clinical trial':ab,ti OR randomized:ab,ti OR placebo:ab,ti OR 'clinical trials as topic':ab,ti OR randomly:ab,ti OR trial:ab,ti OR prospective:ab,ti | 3119832 |
| 5 | 1 AND 2 AND 3 AND 4 | 1360 |

| Cocrhance | | |
| --- | --- | --- |
| No. | Query | Results |
| 1 | (Urinary Bladder Neoplasms OR Neoplasm, Urinary Bladder OR Urinary Bladder Neoplasm OR Bladder Tumors OR Bladder Tumor OR Tumor, Bladder OR Tumors, Bladder OR Neoplasms, Bladder OR Bladder Neoplasms OR Bladder Neoplasm OR Neoplasm, Bladder OR Urinary Bladder Cancer OR Cancer, Urinary Bladder OR Malignant Tumor of Urinary Bladder OR Cancer of the Bladder OR Bladder Cancer OR Bladder Cancers OR Cancer, Bladder OR Cancer of Bladder OR urothelial carcinoma OR urothelial cancer):ab,ti,kw | 7173 |
| 2 | (immunotherapy OR immune checkpoint inhibitor OR PD-1 inhibitor OR PD-L1 inhibitor OR Atezolizumab OR Nivolumab OR Durvalumab OR Pembrolizumab OR Avelumab OR Erdafitinib OR Enfortumab Vedotin OR Sacituzumab Govitecan OR Nectin-4 OR disitamab vedotin OR FGFR):ti,ab,kw | 21384 |
| 3 | (first-line OR untreated OR initial OR primary OR newly-diagnosed OR newly):ti,ab,kw | 732711 |
| 4 | (randomized controlled trial OR controlled clinical trial OR Randomized OR placebo OR clinical trials as topic OR randomly OR Trial OR Prospective OR Retrospective):ti,ab,kw | 1552667 |
| 5 | 1 AND 2 AND 3 AND 4 | 617 |
