# Supplementary material for: Efficacy and safety of first-line immunotherapy-containing regimens compared with chemotherapy for advanced or metastatic urothelial carcinoma: a network meta-analysis of randomized controlled trials
Source: Front Oncol. 2024 Dec 11;14:1453338. doi: 10.3389/fonc.2024.1453338 (PMC11668658; doi:10.3389/fonc.2024.1453338)
Supplement: Supplementary file 2 [file Table2.docx]

**Pooled HR derived from the network meta-analysis examining the relationship between regimen and OS in mUC patients with high PD-L1 status**

| **Atezo** | . |  |  |  |  |  |  |  |
| --- | --- | --- | --- | --- | --- | --- | --- | --- |
| 0.96 (0.74- 1.26) | **Atezo + Chemo** |  |  |  |  |  |  |  |
| 0.85 (0.69- 1.03) | 0.88 (0.73- 1.05) | **Chemo** |  |  |  |  |  |  |
| 0.89 (0.71- 1.11) | 0.92 (0.75- 1.13) | 1.05 (0.95- 1.16) | **Durva** |  |  |  |  |  |
| 0.96 (0.77- 1.20) | 1.00 (0.81- 1.23) | 1.14 (1.03- 1.26) | 1.08 (0.94- 1.24) | **Durva + Chemo** |  |  |  |  |
| 1.15 (0.91- 1.46) | 1.20 (0.96- 1.49) | 1.36 (1.20- 1.55) | 1.30 (1.11- 1.52) | 1.20 (1.02- 1.40) | **EV + Pembro** |  |  |  |
| 0.96 (0.75- 1.23) | 0.99 (0.79- 1.26) | 1.13 (0.97- 1.32) | 1.08 (0.90- 1.29) | 0.99 (0.83- 1.19) | 0.83 (0.68- 1.01) | **Nivol + Chemo** |  |  |
| 0.84 (0.67- 1.06) | 0.87 (0.71- 1.08) | 1.00 (0.89- 1.12) | 0.95 (0.81- 1.10) | 0.87 (0.75- 1.02) | 0.73 (0.62- 0.87) | 0.88 (0.73- 1.06) | **Pembro** |  |
| 0.89 (0.70- 1.12) | 0.92 (0.74- 1.14) | 1.05 (0.93- 1.18) | 1.00 (0.86- 1.16) | 0.92 (0.79- 1.07) | 0.77 (0.65- 0.91) | 0.92 (0.76- 1.12) | 1.05 (0.89- 1.24) | **Pembro + Chemo** |
| **Pooled HR (95% credible interval) derived from network meta-analysis**  **Coloured indicates a statistically significant comparison.** | | | | | | | | |
